# Recent Insights into the Rhythmogenic Core of the Locomotor CPG

**DOI:** 10.3390/ijms22031394

**Published:** 2021-01-30

**Authors:** Vladimir Rancic, Simon Gosgnach

**Affiliations:** Department of Physiology, University of Alberta, 3-020D Katz Building, Edmonton, AB T6G 2E1, Canada; rancic@ualberta.ca

**Keywords:** spinal cord, locomotion, CPG, rhythm generation

## Abstract

In order for locomotion to occur, a complex pattern of muscle activation is required. For more than a century, it has been known that the timing and pattern of stepping movements in mammals are generated by neural networks known as central pattern generators (CPGs), which comprise multiple interneuron cell types located entirely within the spinal cord. A genetic approach has recently been successful in identifying several populations of spinal neurons that make up this neural network, as well as the specific role they play during stepping. In spite of this progress, the identity of the neurons responsible for generating the locomotor rhythm and the manner in which they are interconnected have yet to be deciphered. In this review, we summarize key features considered to be expressed by locomotor rhythm-generating neurons and describe the different genetically defined classes of interneurons which have been proposed to be involved.

## 1. Introduction

Central pattern generators (CPGs) are neural networks that are capable of generating intrinsic patterns of rhythmic activity independent of descending or sensory inputs. CPGs were first postulated to exist in the spinal cord and control locomotor behaviour a century ago when it was demonstrated that rhythmic central circuits in the spinal cord of decerebrate, deafferented cats were capable of producing alternating contractions of hindlimb flexor and extensor muscles [1]. There has been a great deal of investigation into the detailed structure and mechanism of function of locomotor CPGs in the years since, and it is now accepted that these circuits, which are located in the caudal spinal cord, are capable of generating rhythmic, locomotor-like motor patterns in all mammals, including humans [2,3,4,5,6,7,8]. Spinal cord injury often compromises one’s ability to maintain normal posture, execute coordinated movements, and walk. Much of the research attempting to promote functional recovery of walking following spinal cord injury involved attempts to restore the connectivity of neurons across the injury site [9] to the locomotor CPG, so as to take advantage of its powerful rhythm-generating capacity. Clearly, a detailed characterization of the interneurons that comprise the locomotor CPG and a better understanding of how they are activated and interconnected as a network will assist the development of strategies aimed at enhancing recovery of motor function after spinal cord injury.

Although the mammalian locomotor CPG was discovered more than a century ago, progress towards identifying the interneuronal components of this neural network has been relatively slow [7]. Historically, this could be attributed to the large number of neurons present in the mammalian spinal cord combined with the fact that cells with a similar function are intermingled with other functionally unrelated neurons. Recent advances in molecular genetics, anatomical tracing, and imaging techniques have generated a substantial amount of new information regarding components of the mammalian locomotor CPG, as well as the manner in which these components are controlled by descending input. Elegant electrophysiological and tracing techniques, primarily carried out in the mouse, have revealed the discrete brainstem sites that relay descending commands to the caudal spinal cord and are responsible for starting [10,11], stopping [12], and modulating [13,14] the speed of rhythmic activity produced by the locomotor CPG. At the spinal level, a molecular approach has been used to divide the developing neural tube of the embryonic mouse into ten distinct populations of interneurons (dI1–dI6, V0–V3) based on transcription factor expression [15]. Since transcription factor expression dictates neuronal characteristics such as cell fate, channel composition, axonal projection pattern, and neurotransmitter phenotype, it was originally postulated that populations of neurons with a similar genetic background would have similar characteristics and a similar function during locomotor activity. Studies performed over the past 20 years have characterized each of the genetically defined neuronal populations that settle in the spinal cord and defined their function during locomotor activity. Based on this work, we can now identify the specific populations that are responsible for such key functions as left–right [16,17] and flexor–extensor [18,19] coordination.

Despite this progress, a single genetically defined population of spinal interneurons that is necessary and sufficient for generating rhythmic locomotor activity in mammals has yet to be identified. Since these neurons are responsible for the basic rhythmicity in the spinal cord and their level of excitability dictates the speed of stepping, their identification and anatomical/electrophysiological characterization are necessary if we are to comprehensively understand how mammalian locomotor activity is generated and if we hope to develop effective strategies to activate these neural circuits in a controlled fashion following spinal cord injury. Substantial effort has thus gone into identifying the specific genetically defined interneuronal population responsible for generating the locomotor rhythm (reviewed in [20,21]). While there has been progress in the identification of interneuronal populations responsible for rhythm generation in aquatic species [22,23,24,25], we still know little regarding the identity of these cells in mammals. After a decade of study, in which each population was silenced or ablated without affecting the ability of the network to generate the locomotor rhythm, the emergent view is that the neurons responsible for rhythm generation cannot be encapsulated within any one of the genetically defined interneuronal populations and that an alternate approach must be used if these neurons are to be identified [7,26,27].

Here, we discuss the network mechanisms that have been proposed to account for locomotor activity and review recent work which has revealed key characteristics of neurons likely to be involved in locomotor rhythm generation. Finally, we describe the different classes of interneurons which have been proposed to be involved in this function and focus on identifying the specific features that they possess.

## 2. Conceptual Models of the Locomotor CPG

Since initial work indicating that a neural network situated in the spinal cord is likely to be responsible for the rhythmic alternation characteristic of locomotor activity, several network models varying greatly in complexity have been put forth to account for rhythm generation. The first of these, the half-centre model, contains two rhythm-generating modules on either side of the spinal cord: one that generates rhythmic bursting in flexor motoneurons and one for extensor motoneurons [1,28]. These rhythmogenic modules were predicted to comprise excitatory interneurons with ipsilateral connections onto (a) agonist motoneurons (i.e., the flexor half-centre would contact flexor motoneurons, and the extensor half-centre would contact extensor motoneurons); (b) inhibitory interneurons which inhibit the antagonist motor pools; and (c) inhibitory neurons which synapse onto the antagonist rhythm-generating module. While early studies demonstrated that at least a portion of the interneurons involved in rhythm generation was situated in lamina VII of the lumbar spinal cord in cats [28], precise localization of the rhythm generators and characterization of cellular properties were not revealed.

A subsequent model that was strongly supported by experimental data was the unit burst generator (UBG) model [29]. This built on many of the general principles of the half-centre model but, unlike the half-centre arrangement, was able to account for the observation that all flexors (and all extensors) are not simultaneously activated and inactivated; instead, there is a more nuanced activation pattern and some overlap amongst certain flexor and extensor motor pools. There are also pools of bifunctional motoneurons which burst during both the flexion and extension phases. The UBG model proposes that there are half-centre networks located around each joint, controlling a subset of the flexor and extensor motor pools for each limb. While the principles of rhythm generation were not well defined, it was suggested that mechanisms, such as pacemaker neurons, responsible in other physiological systems/species may be key (see [7] for review).

Recently, the more complex two-layer model of the locomotor CPG was devised which builds on the UBG architecture [30,31]. In this model, the function of rhythm generation during locomotion is clearly separated from pattern formation (activation of motoneurons). This model was developed following the observation of “non-resetting deletions”, in which subsequent bursts of motor pools, following a dropout of activity, occur at the precise time expected had the dropout not taken place [32]. The most plausible explanation to account for this phenomenon was the presence of some higher-level “clock” mechanism which governs locomotor speed but is not directly involved in motoneuron activation. Since this model was devised just as the fields of indelible interneuronal labelling and mathematical/computational modelling were expanding, a number of studies have taken population data from interneurons during locomotion, incorporated this information into a computational model, and the result has been a number of testable hypotheses regarding essential rhythmogenic properties [33,34,35,36]. Many of these properties which have been scrutinized are listed in the following section.

## 3. Intrinsic Properties and Cellular Mechanisms Involved in Locomotor Rhythm Generation

### 3.1. Locomotor Rhythm-Generating Neurons Are Glutamatergic

While many of the mechanisms involved in locomotor rhythm generation, as well as the identity of many of the cells remain unclear, we do know that excitatory neurons, which use the neurotransmitter glutamate, are an essential component of the rhythmogenic core of the mammalian locomotor CPG [37]. Convincing evidence of this comes from experiments in which fictive locomotion was generated in spinal cords isolated from newborn mice and the application of antagonists to the glutamatergic NMDA and/or non-NMDA receptors either slowed, or in some cases, completely abolished rhythmic activity [38,39,40]. Glutamate has also been shown to play an important role in speed regulation as different concentrations of agonists to NMDA and non-NMDA receptors are effective in speeding up and slowing down fictive locomotor activity [41]. Further evidence that glutamatergic neurons are not only involved, but that excitatory neurons situated in the caudal spinal cord are both necessary and sufficient for locomotor rhythm generation, comes from two subsequent studies which incorporated selective optogenetic activation of glutamatergic neurons in the lumbar spinal cord. The first of these studies demonstrated that activation of glutamatergic neurons in this region was sufficient to generate, and maintain, fictive locomotor activity in the isolated spinal cord [42]. A follow-up study took these findings even further by demonstrating that a more discrete pattern of optogenetic stimulation was capable of generating fictive locomotor activity unilaterally, as well as in specific motor pools, while selective activation of inhibitory neurons during a bout of drug-induced fictive locomotion resulted in the cessation of rhythmic activity [43]. In addition to demonstrating the importance of glutamatergic neurons in rhythm generation, the finding that the activation of a restricted region of the spinal cord can generate locomotor activity throughout the lumbar spinal cord provides support for the UBG arrangement of the locomotor CPG.

### 3.2. Ion Channels Involved in Rhythmogenesis

Due largely to the technical inability to access the intermediate laminae of the spinal cord in live tissue for much of the 20th century, a great deal of information on the ion channels and intrinsic electrophysiological properties crucial for rhythm generation has historically been derived from pharmacological studies in mammals and the analysis of locomotor rhythm-generating neurons in other evolutionarily “simpler” species [44,45,46].

The channel perhaps most closely linked with locomotor rhythm generation is the persistent Na+ current (I_NaP_) which is activated at subthreshold voltages and capable of amplifying the response of a neuron to synaptic input. Activation of this channel can result in sustained depolarization and plateau potentials [47], two features considered to be essential during locomotor activity [48,49]. While I_NaP_ was first characterized in the hippocampus [50] and cortex [51], it has also been shown to be present in brainstem neurons and involved in respiratory rhythmogenesis in mammals [52,53,54,55], and there is plenty of evidence indicating ventral interneurons in the lumbar spinal cord express I_NaP_ [56,57,58,59]. Crucially, the application of riluzole (an I_NaP_ antagonist) during a bout of fictive locomotor activity results in the cessation of fictive locomotion in the isolated spinal cord preparation [57,58]. Interestingly, in the two-layer computational model of the locomotor CPG, the neurons which comprise the rhythm-generating core contain only fast Na+, fast K+, and leak channels, in addition to I_NaP_, and this model is able to generate a rhythmic pattern with many of the same features as mammalian fictive locomotion [30,31].

While there is extensive evidence indicating that I_NaP_ is involved in the sustained depolarization of locomotor neurons, work in crustaceans and lampreys has indicated that, in addition to I_NaP,_ other voltage-dependent channels (those activated by the NMDA receptors, as well as L-type Ca^2+^ channels) may also play a role in the depolarization phase of a locomotor burst and remain open until a hyperpolarization mechanism is activated. In locomotor-related neurons, this hyperpolarization is thought to be mediated by a build-up of Ca^2+^ ions inside cells and subsequent activation of K_Ca_ channels [5,60]. Hyperpolarization in turn activates post-inhibitory rebound mechanisms such as the hyperpolarization activated current (I_h_), which leads to slow depolarization, the activation of the voltage-dependent mechanisms responsible for another burst, and eventually, sustained oscillation.

### 3.3. Gap Junctions Are Involved in Locomotor Rhythm Generation

Given the distributed architecture of the network models currently thought to be involved in generating locomotor activity, it stands to reason that a substantial number of rhythm-generating neurons need to be simultaneously recruited/activated. One feature proposed to facilitate this is mutual excitation via electrical synapses. Gap junctions have been found to contribute to rhythmic oscillations and neuronal synchrony in CPGs in many other species [61,62,63,64,65,66] and have been shown to act as a low-pass frequency filter [67,68], which enables slow fluctuations of membrane voltage to pass more effectively between cells.

Evidence of electrotonic coupling has been extensively reported in the spinal cord of neonatal rodents [69,70,71,72], and gap junctions have been observed in adults [71,73,74], although the precise distribution of the gap junctions is not completely understood. The importance of gap-junction coupling in the production of locomotor activity at early time points comes from the findings that a selective block of gap junctions with carbenoxolone disrupts appropriate alternation [75], can slow the frequency of [76], and can completely abolish [72] drug-induced fictive locomotion evoked in neonates. Furthermore, robust locomotor-like activity can be generated in the isolated neonatal spinal cord without action potentials, relying solely on electrotonic coupling [72,77], indicating that gap junctions are likely to be highly organized amongst the rhythm-generating network. However, an apparent decline in gap junctions has been reported after postnatal day 7 in rodents [78], suggests they may play a reduced role in overground stepping.

Interestingly, chemical synaptic neuronal activity is capable of profoundly affecting the neuronal synchronization mediated via electrotonic coupling. In the inferior olive structure, it was shown that activation of NMDA receptors can strengthen the efficacy of gap-junction coupling and promote neuronal synchronization [79]. Activity of I_NaP_ has also been shown to impact gap-junction coupling and, in the thalamic reticular nucleus, can cause up to a four-fold change in electrical synaptic efficacy between neurons [80]. Given the key role of NMDA receptors and I_NaP_ in rhythm generation, a similar modulation of electrotonic coupling in the spinal cord could potentially promote neuronal synchronization at different frequencies of oscillation.

## 4. Molecularly Defined Interneuronal Populations that May Generate the Locomotor Rhythm

Around the turn of the century, pioneering work from the Jessell and Goulding laboratories incorporated a molecular approach to divide the developing spinal cord into a restricted number of genetically distinct neuronal populations based on the transcription factors they express at early embryonic time points [15,81]. By using a variety of techniques to indelibly label, reversibly silence, or ablate these populations, substantial insight has been provided into those populations which are involved in locomotion as well as their specific function. There are some caveats with these studies, including, but not limited to, a lack of clarity with respect to the proportion of a given population that participates in locomotor activity, and the fact that upon closer inspection, each “parent” population can be broken down into an increasing number of somewhat diverse subpopulations [82,83]. Despite this, the incorporation of a molecular approach has enabled us to provide substantial insight into the network structure and mechanism of function of the locomotor CPG.

Although all interneuronal populations of a common genetic lineage situated in the ventral spinal cord postnatally (V0–V3 interneurons as well as Shox2, and Hb9-expressing neurons) have now been silenced or ablated, the locomotor rhythm is remarkably persistent and has yet to be abolished. This led to the suggestion that rather than relying on a single interneuronal population, locomotor rhythm generation relies on subsets of multiple populations [7,26,27]. Thus far, the goal when investigating populations that may be involved in the rhythm generating circuitry has been to focus on excitatory interneuronal populations, determine whether they express properties characteristic of rhythm generators, and investigate whether the locomotor rhythm is altered in their absence, with a change in the locomotor frequency likely indicating that they are part of the “clock” in the two-layer model, and involved in rhythmogenesis. In this section, we identify the interneuronal populations that have been predicted to be involved and review the features of each population that supports locomotor rhythm generation. In Table 1, we present each excitatory neuronal population found in the mammalian spinal cord postnatally and identify those with properties consistent with a role in rhythm generation. Note the lack of data regarding some features such as rhythmic activity during locomotor activity and electrophysiological properties. They are largely due to a difficulty accessing specific subpopulations for intracellular recordings.

### 4.1. Hb9-Expressing Interneurons

Expression of the homeobox transcription factor Hb9 can be seen in the spinal cord by embryonic day 9 (E9) and was initially thought to be a selective marker of motoneurons [3]. Subsequent to this, a small population of ipsilaterally projecting spinal interneurons situated medially, just ventral to the central canal, primarily in the T13 to L2/L3 segments, was also shown to express this transcription factor [26,75,84,85,86].

While this population of cells is exclusively excitatory, a comprehensive characterization of Hb9 interneurons used two transgenic mouse strains (Hb9^GFP^ and Hb9^Cre^). Each strain labelled a larger population of neurons with only a subset of labelled cells expressing the Hb9 protein. Approximately one third of this larger population was shown to be excitatory, and two thirds inhibitory [26,84]. Although some Hb9-expressing interneurons synapse onto motoneurons [87], a subset have been shown to interconnect with one another, as well as non-Hb9-expressing neurons, via gap-junction coupling [70,75]. Electrophysiological characterization of this population was carried out solely on the glutamatergic Hb9-expressing interneurons and indicated that 86% of this population is rhythmically active during fictive locomotor activity [75,84,85] and that much of the population exhibits intrinsic properties believed to support endogenous bursting, including I_NaP_ [57,59,86]. Based on this preliminary characterization, it was hypothesized that Hb9-expressing cells are involved in rhythm generation [59,84,86,88]; however, a subsequent study indicated that rhythmic spiking activity in Hb9-expressing cells typically occurs more than a second after the onset of ipsilateral motor activity [85]. Since logic would dictate that activity in rhythm-generating neurons would precede that in motoneurons, this finding brought their specific rhythmogenic role into question.

One of the primary challenges when attempting to definitively determine the function of these interneurons during locomotion is that Hb9 is also expressed in motoneurons, and thus, experiments in which these neurons are silenced or ablated (and the resultant locomotor pattern is analyzed) were unable to be carried out. In an attempt to circumvent this issue, a mouse model was generated in which synaptic transmission in glutamatergic Hb9 neurons alone was arrested [26]. This enabled their putative role in rhythm generation to be assessed, as it would be assumed to be carried out by the glutamatergic subset, while inhibitory Hb9-expressing cells and motoneurons (which are primarily cholinergic) would be unaffected in this mouse model. Experiments in spinal cords taken from these animals indicated that the frequency of fictive locomotion was consistently slower in the absence of glutamatergic Hb9 interneurons, with left–right and flexor–extensor coordination unperturbed, suggesting that the excitatory Hb9-expressing interneurons may in fact be part of the locomotor rhythm-generating network [26], and the Hb9-expressing neurons shown to have a delayed activation may simply not have been part of the excitatory rhythm-generating subset. Importantly, the mouse model used for these experiments is not viable beyond P0, and thus, the role of Hb9 interneurons in locomotor rhythm generation in older animals is unknown.

### 4.2. Shox2-Expressing Interneurons

The transcription factor Shox2, which is co-expressed with Hb9 in some neurons [26], is first expressed in the spinal cord at E11.5 and can still be seen in approximately 35% of cells at P0-P1, primarily in the intermediate zone, throughout the rostrocaudal extent of the spinal cord [27]. Postnatally, approximately 77% of Shox2-expressing interneurons co-express the transcription factor Chx10, which defines the V2a interneuronal population [27,89,90], an overwhelmingly excitatory group of neurons. Although V2a cells are involved in locomotor rhythmogenesis in zebrafish [22,23,24,25], in mice they are involved in the speed-dependent regulation of left–right alternation [89]. More than 98% of neurons expressing Shox2 are glutamatergic, and axons typically project ipsilaterally [27]. Shox2-expressing interneurons have been shown to contact ipsilateral motoneurons, commissural interneurons, and other Shox2+ interneurons located in the same spinal segment [27]. Connectivity between Shox2-expressing neurons can be unidirectional or bidirectional, with unidirectional connections occurring rarely, and mediated mainly by chemical synapses. Bidirectional connections, which are much more common, are primarily mediated by electrical coupling [76]. Given their developmental similarity to the V2a interneuronal population, the connectivity of those Shox2-expressing cells that were Chx10+ and Chx10 was further investigated. Interestingly, Shox2+/Chx10- cells were regularly found to contact one another via electrical synapses but did not contact Shox2+/Chx10+ cells. [76].

Although a difficulty distinguishing between the Shox2-expressing neurons which are Chx10+ and Chx10- has thus far precluded a thorough electrophysiological analysis, or detailed description of the distribution of the Shox2+/Chx10- neurons, intracellular recordings of the Shox2 population as a whole indicates that 70% of these neurons are rhythmically active during fictive locomotion. Intrinsic electrophysiological properties of Shox2-expressing neurons have yet to be assessed, so it is unclear whether they may be capable of endogenous bursting. Due to the overlap of Chx10 and Shox2, it has not yet been possible to solely ablate those neurons which express Shox2+ alone and investigate locomotor activity in their absence. However, ablation of the entire Shox2-expressing population drastically reduced the frequency of locomotor outputs, while ablation of just the subpopulation which express both Shox2 and Chx10 had no effect on the locomotor rhythm [27]. Based on these data, it was inferred that those Shox2+ neurons that did not co-express Chx10 are involved in locomotor rhythm generation, likely via a mechanism relying heavily on electrical synapses [76].

It is important to note that while gap-junction coupling between Shox2+ cells was detected in early postnatal stages, the strength of the electrotonic coupling progressively decreases with the maturation of the spinal cord [76]. The rhythmogenic role of Shox2-expressing interneurons in more mature preparations has yet to be investigated; however, this finding raises the possibility that Shox2-expressing interneurons may be involved in rhythm generation at early developmental time points, although the reliance on these cells may diminish over time.

## 5. Conclusions

Despite a tremendous amount of recent progress in identifying interneuronal components of the locomotor CPG, as well as characteristics of rhythm-generating neurons, a population of spinal interneurons that is necessary and sufficient for generating rhythmic locomotor activity has yet to be pinned down. There is compelling evidence that at least two genetically defined interneuronal populations are involved in locomotor rhythm generation, although it is possible that a restricted subset of several additional populations may be involved.

Finer grain investigation with cutting-edge techniques continues to reveal an increasing number of subsets within each parent population [91]. The more we learn about each subset, the greater the chances we have of identifying additional populations involved in rhythmogenesis. Take, for example, the V3 interneuronal population. Initial analysis indicated that this excitatory population primarily synapses onto contralateral motoneurons [92], ruling out a role in locomotor rhythm generation. Recent, more detailed, analyses have revealed that this is an extremely diverse population of cells [93,94] with a subset located in the ventromedial region of the spinal cord projecting to other interneurons (V3 and non-V3) on the ipsilateral side of the spinal cord [95]. Furthermore, electrophysiological analysis of V3 neurons in this region indicates that they do possess several electrophysiological properties of endogenous oscillators, including post-inhibitory rebound and the ability to oscillate intrinsically in high concentrations of NMDA [93,94]. Is it possible that this medial subset of V3 cells is part of the locomotor rhythm generating network? Ablation of the entire V3 population results in an unbalanced locomotor rhythm, but given the diversity of V3 cells, it is necessary to ablate the V3_VMED_ subset individually to assess a possible role in rhythmogenesis. Given the predicted importance on electrical synapses in locomotor rhythm generation, it is likely that components involved in this function are located in close proximity to one another. V3_VMED_ neurons certainly fit this criterion, as they are situated close to the central canal along with the Hb9 cells and a portion of the Shox2 neurons. In addition, it has been demonstrated that direct stimulation of ventral roots [96] or motoneurons [97] results in fictive locomotion in neonatal mice. These data indicate that motoneurons may be involved in rhythm generation, presumably via glutamatergic synapses onto components of the locomotor CPG. Given that subsets of V3 neurons have been shown to receive input from motoneurons, it is possible that they are involved in facilitating locomotor rhythmogenesis via this recurrent pathway [95].

What is very clear is that at this point in time, there is no specific transcription factor that is unique to the rhythm-generating neurons, and the identification of the rhythmogenic core of the locomotor CPG will likely require an approach that focuses on a thorough characterization of intrinsic properties, and the specific activity pattern, of neurons belonging to several interneuronal populations during locomotor activity. Despite these challenges, the identification and characterization of these cells is necessary if we are to understand how mammalian locomotion is generated and if we hope to develop effective strategies to activate these circuits in a controlled fashion following spinal cord injury.

## Figures and Tables

**Table 1:** Rhythmogenic features of genetically-defined excitatory interneurons in the ventral spinal cord.

Neuronal Subset	Rhythmic Activity during Locomotion	Axonal Projection	I_NaP_	Gap Junctions	Locomotor Phenotype When Population Is Missing
V0_V_	?	contralateral	?	?	L/R alternation defects at high speed
V2_A_	?	ipsilateral to MNs and V0 cells	?	Y	L/R alternation defects at high speed
Shox2+	Yes	ipsilateral to Shox2+ cells	?	Y	Decrease in locomotor frequency
V3_VMED_	minimal (c-fos)	ipsilateral to V3_VMED_, V3_VLAT_	?	Y	Unbalanced rhythm.
V3_VLAT_	Yes (c-fos)	ipsilateral/ contralateral MNs	?	?	
V3_D_	Yes (c-fos)		?	?	
Hb9+	Yes	ipsilateral to Hb9, and other interneurons as well as MNs.	Y	Y	Decrease in locomotor frequency

## Data Availability

Not Applicable.
